# *rasbhari*: Optimizing Spaced Seeds for Database Searching, Read Mapping and Alignment-Free Sequence Comparison

**DOI:** 10.1371/journal.pcbi.1005107

**Published:** 2016-10-19

**Authors:** Lars Hahn, Chris-André Leimeister, Rachid Ounit, Stefano Lonardi, Burkhard Morgenstern

**Affiliations:** 1 University of Göttingen, Department of Bioinformatics, Göttingen, Germany; 2 University of California, Riverside, Department of Computer Science and Engineering, Riverside, California, United States of America; 3 University of Göttingen, Center for Computational Sciences, Göttingen, Germany; UCSD, UNITED STATES

## Abstract

Many algorithms for sequence analysis rely on word matching or word statistics. Often, these approaches can be improved if binary patterns representing *match* and *don’t-care* positions are used as a filter, such that only those positions of words are considered that correspond to the *match positions* of the patterns. The performance of these approaches, however, depends on the underlying patterns. Herein, we show that the *overlap complexity* of a pattern set that was introduced by Ilie and Ilie is closely related to the *variance* of the number of matches between two evolutionarily related sequences with respect to this pattern set. We propose a modified hill-climbing algorithm to optimize pattern sets for database searching, read mapping and alignment-free sequence comparison of nucleic-acid sequences; our implementation of this algorithm is called *rasbhari*. Depending on the application at hand, *rasbhari* can either minimize the *overlap complexity* of pattern sets, maximize their *sensitivity* in database searching or minimize the *variance* of the number of pattern-based matches in alignment-free sequence comparison. We show that, for database searching, *rasbhari* generates pattern sets with slightly higher sensitivity than existing approaches. In our *Spaced Words* approach to alignment-free sequence comparison, pattern sets calculated with *rasbhari* led to more accurate estimates of phylogenetic distances than the randomly generated pattern sets that we previously used. Finally, we used *rasbhari* to generate patterns for short read classification with *CLARK-S*. Here too, the sensitivity of the results could be improved, compared to the default patterns of the program. We integrated *rasbhari* into *Spaced Words*; the source code of *rasbhari* is freely available at http://rasbhari.gobics.de/

## Author Summary

We propose a fast algorithm to generate spaced seeds for database searching, read mapping and alignment-free sequence comparison. Spaced seeds—i.e. patterns of match and don’t-care positions—are used by many algorithms for sequence analysis; designing optimal seeds is therefore an active field of research. In sequence-database searching, one wants to optimize sensitivity, i.e. the probability of finding a region of homology; this can be done by minimizing the so-called overlap complexity of pattern sets. In alignment-free DNA sequence comparison, the number *N* of pattern-based matches is used to estimate phylogenetic distances. Here, one wants to minimize the variance of *N* in order to obtain stable phylogenies. We show that for spaced seeds, the overlap complexity—and therefore the sensitivity in database searching—is closely related to the variance of *N*. Our algorithm can optimize the sensitivity, overlap complexity or the variance of *N*, depending on the application at hand.

## Introduction

*k*-mers, i.e. words of length *k*, are used in many basic algorithms for biological sequence comparison. Word matches are used, for example, as *seeds* in the *hit-and-extend* approach to database searching and read mapping [1–3]. Here, fast algorithms are applied to find pairs of identical or similar words between two sequences. A slower but more sensitive alignment method is then used to extend these word pairs to both directions, to distinguish biologically relevant homologies from spurious word matches. In alignment-free sequence comparison, sequences are often represented by word-frequency vectors to estimate distances or similarities between them, e.g. as a basis for phylogeny reconstruction [4–8], see [9] for a review. Similarly, word statistics are used to classify DNA or protein sequences [10–12], for datamining [13] and for remote homology detection [14].

It is well known that many word-based approaches produce better results if *spaced words* or *seeds* are used instead of the previously used *contiguous* words or word matches. That is, for a pre-defined binary pattern *P* representing *match* and *don’t-care* positions, one considers only those positions in a word of the same length that correspond to the *match* positions of *P*. Pattern-based word matching has been proposed for *hit-and-extend* database searching by Ma et al. [15], see also [16]. Spaced seeds are also routinely used in metagenome sequence clustering and classification [17, 18], protein classification [19], read mapping [20, 21], to find *anchor points* for multiple sequence alignment [22, 23] and for alignment-free phylogeny reconstruction [24]. Similarly, the *average common substring* approach to sequence comparison [25] could be improved by allowing for mismatches [26–30]. Brejova et al. extended the concept of *spaced seeds* to homologies among protein-coding regions [31] and introduced *vector seeds* [32]. In general, the advantage of pattern-based approaches is the fact that spaced-word occurrences at neighbouring sequence positions are statistically less dependent than occurrences of contiguous words [33, 34]. Often *sets* of patterns are used, instead of single patterns; such *multiple* spaced seeds are now a standard filtering step in homology searching [35, 36].

In pattern-based approaches, the underlying patterns of match and don’t-care positions are of crucial importance for the quality of the results. Generally, non-periodic patterns are preferred since they minimize redundancies between overlapping words or word matches and lead to a more even distribution of matches. This increases the probability of obtaining a hit between two homologous sequences in database searching and leads to more stable distance estimates in phylogeny reconstruction. Noé and Martin [37] defined a *coverage criterion* for multiple spaced seeds and showed that this criterion is related to the *Hamming distance* between two sequences. In the context of database searching, patterns or sets of patterns are often called *seeds*. (Originally, the word *seed* denoted a match of—contiguous or spaced—words between a query and a database sequence that would be extended to the left and to the right. But now *seed* often denotes the underlying pattern in pattern-based approaches).

In hit-and-extend database searching, the *sensitivity* of a pattern set is defined as the probability of finding at least one hit within a gap-free alignment of a given length *L* and probability *p* for a match between two residues. Since each hit is extended to a local alignment, the sensitivity is the proportion of homologies that will be found by a search program—under the above simple model of homology, and under the assumption that each extension of a hit in a homologous region will verify the homology. In database searching, the goal is thus to maximize the sensitivity of pattern sets.

Calculating the sensitivity of a pattern set is *NP-hard* [33]. The sensitivity can be approximated by dynamic programming [15, 38], but the run time of this algorithm is still exponential in the length of the pattern. In *PatternHunter II*, a *greedy* algorithm is used to find suitable patterns. In 2007, Ilie and Ilie introduced the *overlap complexity* of a pattern set and showed experimentally that—for a given number of patterns with a given length and number of match positions—minimizing the overlap complexity corresponds to maximizing the sensitivity in database searching [39]. In contrast to the sensitivity, however, the overlap complexity can be easily calculated. To find optimal pattern sets, Ilie and Ilie proposed a *hill-climbing algorithm* that minimizes the overlap complexity. They implemented their algorithm in a software tool called *SpEED* [40], which is several orders of magnitude faster than competing approaches and is now considered the state-of-the-art in seed optimization.

Recently, we proposed to use *spaced-word* frequencies instead of word frequencies for alignment-free sequence comparison [24, 41]. We showed that phylogenetic trees calculated from spaced-word frequencies are more accurate than trees calculated from contiguous-word frequencies. As in database searching, our results could be improved by using *multiple* patterns. In our original study, we used randomly generated multiple patterns of *match* and *don’t-care* positions. In a follow-up paper, we studied the number *N* of spaced-word matches between two DNA sequences for a set of binary patterns [34]. Our data suggest that minimizing the variance of *N* for pattern sets improves alignment-free phylogeny reconstruction.

In this paper, we first show that the variance of the number *N* of spaced-word matches is closely related to the *overlap complexity* of the underlying set of patterns. We propose a modified hill-climbing algorithm that can be used to generate pattern sets, either with minimal variance of *N*, or with minimal overlap complexity, or with maximal sensitivity in database searching, depending on the application at hand. While the algorithm proposed in [39] iterates over all patterns *P* in a set P of patterns and all pairs of positions in *P* to improve P, we calculate for each pattern P∈P how much *P* contributes to the variance or overlap complexity, respectively, of P. We then modify those patterns first that contribute most to the variance or complexity.

The implementation of our approach is called *rasbhari (Rapid Approach for Seed optimization Based on a Hill-climbing Algorithm that is Repeated Iteratively)*. Experimental results show that pattern sets calculated with *rasbhari* have a slightly higher sensitivity in database searching than pattern sets calculated with *SpEED*, while the run time of both programs is comparable. In alignment-free sequence comparison, we obtain more accurate phylogenetic distances if we use *rasbhari* to minimize the variance of *N* for the underlying pattern sets, than we obtained with the randomly generated pattern sets that we previously used. In a third application, we used pattern sets generated with *rasbhari* in the program *CLARK-S* [18] for short read classification. The sensitivity of the classification could be improved in this way, while *rasbhari* is substantially faster than the method that is used by default for pattern generation in *CLARK-S*.

A earlier version of this paper has been published at the preprint server *arXiv* [42].

## Methods

### Overlap complexity

We consider sets $P=\{P_{1},\ldots ,P_{m}\}$ of binary patterns, where *ℓ*_*r*_ is the length of pattern *P*_*r*_ and *ℓ* = max_*r*_ *ℓ*_*r*_. That is, each *P*_*r*_ is a word of length *ℓ*_*r*_ over the alphabet {1, 0}. A ‘1’ in a pattern *P*_*r*_ represents a *match* position, a ‘0’ a *don’t-care* position. For a single pattern *P*_*r*_, the number of match positions is called its *weight* *w*. For simplicity, we assume that all patterns in a set P have the same weight.

In [34], we considered for two patterns *P*_*r*_, *P*_*r*′_ and s∈Z the number *n*(*P*_*r*_, *P*_*r*′_, *s*) of positions that are match positions of *P*_*r*_ *or* match positions of *P*_*r*′_ (or both), if *P*_*r*′_ is shifted by *s* positions to the right, relative to *P*_*r*_. If *s* is negative, *P*_*r*′_ is shifted to the left. For *P*_*r*_ = 101011, *P*_*r*′_ = 111001, for example, if *P*_*r*′_ is shifted by 2 positions to the right, relative to *P*_*r*_, then there are 6 positions (marked by asterisks below) that are match positions of *P*_*r*_ or *P*_*r*′_. Thus, for *s* = 2, we have *n*(*P*, *P*_*r*′_, 2) = 6:

$$\begin{array}{cccccccccc} P_{r}: & & 1 & 0 & 1 & 0 & 1 & 1 & & \\ P_{r^{\prime }}: & & & & 1 & 1 & 1 & 0 & 0 & 1\\ & & * & & * & * & * & * & & *\\ & & & & \$ & & \$ & & & \end{array}$$

For the same situation, Ilie and Ilie [39] defined *σ*[*s*] = *σ*_*r*,*r*′_[*s*] as the number of positions where *P*_*r*_ *and* *P*_*r*′_ have a match positions, such as the positions marked by ‘$’ above. In the above example one would therefore have *σ*[2] = 2. The *overlap complexity (OC)* of a set of patterns $P=\{P_{1},\ldots ,P_{m}\}$ is then defined in [39] as

$$\sum_{r\leq r^{\prime }}\sum_{s=1-\ell_{r^{\prime }}}^{\ell_{r}-1}2^{\sigma_{r,r^{\prime }}\left[s\right]}$$ (1)

Note that, since for any two patterns *P*_*r*_, *P*_*r*′_ and s∈Z, the equality

$$\sigma_{r,r^{\prime }}\left[s\right]=2w-n\left(P_{r},P_{r^{\prime }},s\right)$$

holds, the overlap complexity of a set P can be written as

$$\sum_{r\leq r^{\prime }}\sum_{s=1-\ell_{r^{\prime }}}^{\ell_{r}-1}2^{\sigma_{r,r^{\prime }}\left[s\right]}=2^{2w}\cdot \sum_{r\leq r^{\prime }}\sum_{s=1-\ell_{r^{\prime }}}^{\ell_{r}-1}{\left(1/2\right)}^{n(P_{r},P_{r^{\prime }},s)}$$ (2)

Consequently, if we are looking at sets P of *m* patterns with fixed weight *w* and lengths *ℓ*_*r*_, then minimizing the overlap complexity of P is equivalent to minimizing the sum

$$\sum_{r\leq r^{\prime }}\sum_{s=1-\ell_{r^{\prime }}}^{\ell_{r}-1}{\left(1/2\right)}^{n(P_{r},P_{r^{\prime }},s)}$$ (3)

Ilie and Ilie showed experimentally that the *OC* is closely related to the sensitivity of a pattern set. More precisely, they showed that for pattern sets with a given number of patterns of given lengths and weight, minimizing the *OC* practically amounts to maximizing the sensitivity. Consequently, in order to find suitable pattern sets for hit-and-extend approaches in database searching, they proposed to search for pattern sets with minimal *OC*. The main advantage of this approach is the fact that the *OC* of a pattern set is much easier to calculate than its sensitivity.

### Variance of the number of spaced-word matches

For a pattern *P* of length *ℓ*, we say that two sequences *S*_1_ and *S*_2_ have a *spaced-word match* with respect to *P* at (*i*, *j*), if the *ℓ*-mers starting at *i* and *j* have identical characters at all *match* positions of *P*, i.e. if one has *S*_1_(*i* + *π* − 1) = *S*_2_(*j* + *π* − 1) for all match positions *π* in *P*. The sequences below, for example, have a spaced-word match at (2, 4) with respect to the pattern *P* = 110101. Indeed, the 6-mers starting at positions 2 and 4 of the sequences are identical at all positions corresponding to a *match position* (‘1’) in *P*, while positions at *don’t-care positions* (‘0’) may be matches or mismatches.

$$\begin{array}{cccccccccccc} S_{1}: & & & A & A & T & C & G & A & T & C & A\\ S_{2}: & C & G & T & A & T & T & G & A & T & T & \\ P: & & & & 1 & 1 & 0 & 1 & 0 & 1 & & \end{array}$$

In [34], we considered spaced-word matches between two sequences *S*_1_ and *S*_2_ with respect to a set $P=\{P_{1},\ldots ,P_{m}\}$ of patterns, so-called P-matches. Note that there can be up to *m* P-matches at each pair (*i*, *j*) of positions of *S*_1_ and *S*_2_, one P-match for each pattern *P*_*r*_ in P. We studied the number $N=N(S_{1},S_{2},P)$ of P-matches between sequences *S*_1_ and *S*_2_ under a simplified model of evolution without insertions and deletions, with a match probability *p* for pairs of homologous positions and a *background* match probability of *q*. Thus, in our model we have

$$Pr\left(S_{1}\left[i\right]=S_{2}\left[j\right]\right)=\left\{\begin{array}{cc} p & \;\text{if}\;i=j\\ q & \;\text{if}\;i\neq j \end{array}\right.$$

It is easy to see that, for a pattern set P, the *expected* number of P-matches depends only on the number *m* of patterns in P and on their lengths *ℓ*_*i*_ and their weight *w*, i.e. number of match positions, but not on the particular sequence of *match* and *don’t-care* positions in P. The variance of *N*, however, does depend on the sequence of *match* and *don’t-care* positions.

As discussed in [34], many alignment-free distance or similarity measures are—explicitly or implicitly—a function of the number *N* of (spaced) word matches. To obtain stable distance measures for phylogeny reconstruction, it is therefore desirable to use pattern sets with a low variance of *N*. For a given set $P=\{P_{1},\ldots ,P_{m}\}$ of patterns of lengths *ℓ*_1_, …, *ℓ*_*m*_ and weight *w*, and with the above simple model of evolution, the variance of *N* can be approximated by

$$\begin{array}{cc} Var(N)\approx & \left(L-\ell +1\right)\cdot \sum_{r\leq r^{\prime }}\sum_{s\in R(r,r^{\prime })}\left(p^{n(P_{r},P_{r^{\prime }},s)}-p^{2w}\right)\\ & +\;\;\left(L-\ell +1\right)\cdot \left(L-\ell \right)\cdot \sum_{r\leq r^{\prime }}\sum_{s\in R(r,r^{\prime })}\left(q^{n(P_{r},P_{r^{\prime }},s)}-q^{2w}\right) \end{array}$$ (4)

where *L* is the length of *S*_1_ and *S*_2_, respectively, and

$$R\left(r,r^{\prime }\right)=\left\{\begin{array}{cc} \left\{1-\ell_{r^{\prime }},\ldots ,\ell_{r}-1\right\} & \;\text{if}\;r<r^{\prime }\\ \left\{0,\ldots ,\ell_{r}-1\right\} & \;\text{if}\;r=r^{\prime } \end{array}\right.$$

is the range in which *P*_*r*′_ is to be shifted against *P*_*r*_ [34]. Note that for different patterns *P*_*r*′_ ≠ *P*_*r*_ we have to consider all shifts between 1 − *ℓ*_*r*′_ and *ℓ*_*r*_ − 1 of *P*_*r*′_ against *P*_*r*_, for example:

Pr:10111011Pr′:10101,⋯,10101s:-43

By contrast, if a pattern *P*_*r*_ is shifted against itself, only shifts between 0 and *ℓ*_*r*_ − 1 need to be considered, to avoid double counting of shifts, for example:

Pr:10111011Pr:1011,⋯,1011s:03

In [34], we ignored this fact and gave a slightly different estimate for *Var*(*N*).

**Fig 1:**
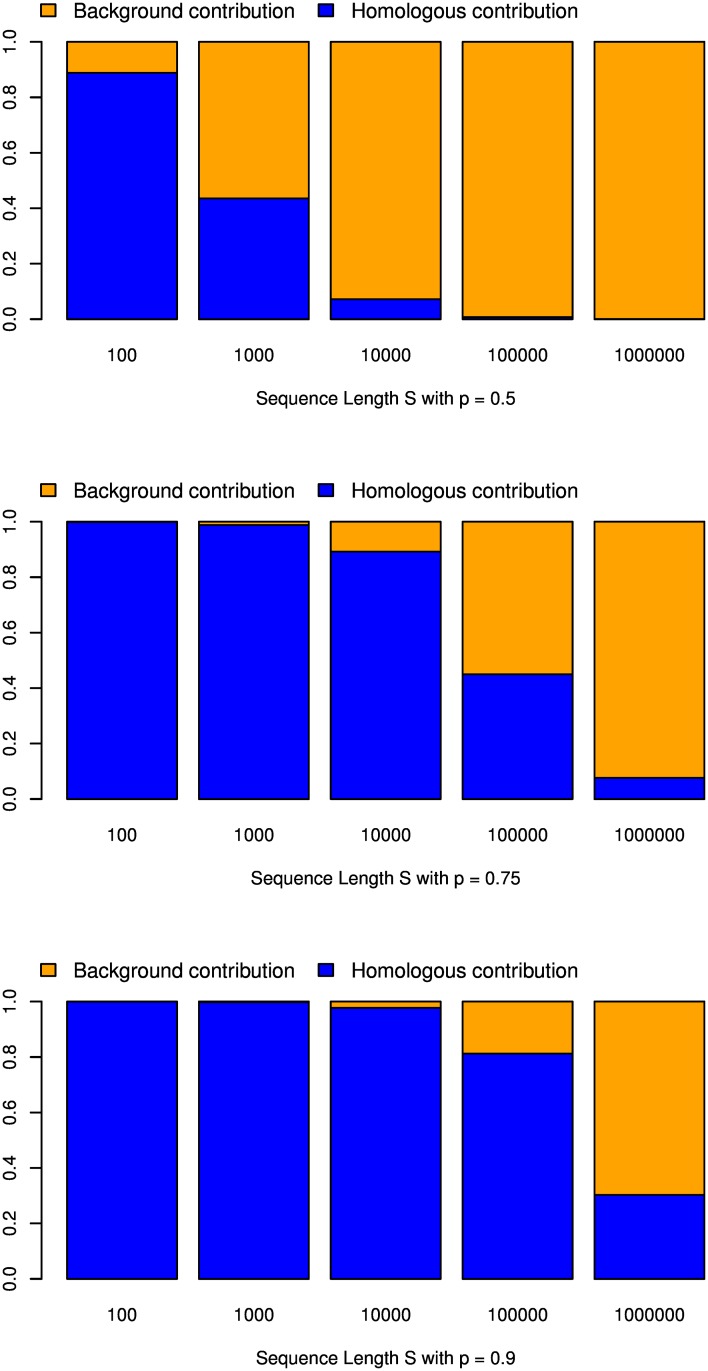
Homolgue and background contribution to the variance of the number N of spaced-word matches. Contribution of the *homologue* and *background* variance to the total variance of the number *N* of spaced-word matches in eq (4) for different match probabilities *p* and sequence lengths *L*.

On the right-hand side of Eq (4), the first summand is the variance of the ‘homologous’ spaced-word matches (in a model without indels, these are spaced-word matches involving the same positions in both sequences), while the second summand comes from background matches. The *relative* weight of the background matches in Eq (4) depends on the match probability *p* and the sequence length *L*; for *p* >> *q* and small *L*, the *Var*(*N*) is dominated by the ‘homologous’ term, see Fig 1. Obviously, for large *L*, the background spaced-word matches dominate the ‘homologous’ ones, since the number of background matches grows quadratically with *L*, while the ‘homologous’ matches grow only linearly.

Note that, for *L*, *ℓ* and *w* fixed, minimizing the *Var*(*N*) amounts to minimizing

$$\sum_{r\leq r^{\prime }}\sum_{s\in R(r,r^{\prime })}p^{n(P_{r},P_{r^{\prime }},s)}\;+\;\;\left(L-\ell \right)\cdot \sum_{r\leq r^{\prime }}\sum_{s\in R(r,r^{\prime })}q^{n(P_{r},P_{r^{\prime }},s)}$$ (5)

Comparison with Eq (2) shows that, in the special case of *p* = 1/2, the first summand of Eq (5) that corresponds to the *homologous* matches is almost identical with the *overlap complexity* defined by Ilie and Ilie (except for the range *R*(*r*, *r*) in which a pattern *P*_*r*_ is shifted against itself). For sequences of moderate length, the overlap complexity can therefore be seen as an approximation to the variance of the number of spaced-word matches.

In any case, the overlap complexity and the *Var*(*N*) for a set of pattern $P=\{P_{1},\ldots ,P_{m}\}$ both have the form

$$\sum_{r\leq r^{\prime }}\alpha_{r,r^{\prime }}\left(P\right)$$ (6)

with

$$\begin{array}{c} \alpha_{r,r^{\prime }}\left(P\right)=\left\{\begin{array}{cc} \sum_{s=1-\ell_{r^{\prime }}}^{\ell_{r}-1}2^{\sigma_{r,r^{\prime }}\left[s\right]} & \left(OC\right)\\ \left(L-\ell +1\right)\sum_{s\in R(r,r^{\prime })}\left(p^{n(P_{r},P_{r^{\prime }},s)}+\left(L-\ell \right)\cdot q^{n(P_{r},P_{r^{\prime }},s)}\right) & \left(Var\right) \end{array}\right. \end{array}$$ (7)

Our optimization problem is therefore: for integers *m*, ℓ_1_, … *ℓ*_*m*_, *w*, find a set P of *m* patterns of lengths *ℓ*_1_, …, *ℓ*_*m*_ and weight *w* that minimizes the sum Eq (6).

### Hill-climbing algorithms to find sets of patterns with minimal *Var*(*N*) or *OC*

Both *SpEED* and our new algorithm start with randomly generated pattern sets and use *hill-climbing* to gradually reduce the *OC* or *Var*(*N*). If one wants to find a pattern set with maximal *sensitivity*, the sensitivity is calculated for the pattern set that is produced by this procedure (this step is omitted, of course, if *rasbhari* is used to minimize *Var*(*N*) or *OC*). The whole procedure is repeated, and the pattern set with the overall highest sensitivity—or lowest *Var*(*N*) or *OC*, respectively—is returned.

#### Original hill-climbing algorithm

To improve the current pattern set P, the hill-climbing algorithm implemented in *SpEED* looks at all triplets (*r*, *i*, *j*) where *P*_*r*_ is a pattern in P, and *i* and *j* are a *match position* and a *don’t-care* position in *P*_*r*_, respectively. For each such triplet (*r*, *i*, *j*), the algorithm considers the pattern set that would be obtained from P by swapping *i* and *j* in *P*_*r*_—i.e. by turning *i* into a don’t-care and *j* into a match position. The *OC* is calculated for all pattern sets that can be obtained in this way, and the one with the lowest *OC* is selected as the next pattern set P. This is repeated iteratively.

There are *O*(*m* ⋅ *ℓ*^2^) triplets (*r*, *i*, *j*) to be considered to modify the current pattern set P. For each of these triplets, the *OC* is to be calculated for the pattern set that would be obtained by swapping *i* and *j* in *P*_*r*_. To this end, the modified pattern *P*_*r*_ has to be compared to the *m* − 1 remaining patterns in P which, for each pattern comparison, involves *O*(*ℓ*) shifts of two patterns against each other. In each shift, the number of common match positions is to be counted, which takes again *O*(*ℓ*) time. Thus, calculating the *OC* of the pattern set obtained by swapping two positions *i* and *j* in a pattern *P*_*r*_ takes *O*(*m* ⋅ *ℓ*^2^) time, so finding an optimal triplet (*r*, *i*, *j*) to determine the next pattern set takes *O*(*m*^2^ ⋅ *ℓ*^4^) time. In *SpEED*, this step is repeated until the *OC* cannot be improved further, i.e. until a local minimum is reached. For the pattern set that is obtained by this hill-climbing routine, the sensitivity is calculated. This whole procedure is repeated 5,000 times, and finally the set with the best sensitivity is returned.

#### Modified hill-climbing algorithm

In our modified hill-climbing algorithm, we also swap a match position *i* with a don’t-care position *j* in some pattern *P*_*r*_ in each step of the algorithm, and we evaluate the *OC* or *Var*(*N*) of the pattern set that would be obtained by this operation. However, instead of looking at *all* possible triplets (*r*, *i*, *j*), we look at those patterns first that contribute most to the *OC* or *Var*(*N*), respectively, of the current pattern set P. The contribution

$$C_{r}=\sum_{r^{\prime }}\alpha_{r,r^{\prime }}$$ (8)

of a pattern $P_{r}\in P$ to the *OC* or *Var*(*N*) of P can be calculated as a by-product, whenever *OC* or *Var*(*N*) is calculated, with *α*_*r*,*r*′_ as in Eq (7). We then sort the patterns in $P_{r}\in P$ according to the values *C*_*r*_, and we process them in descending order of *C*_*r*_, i.e. we look at those patterns first that contribute *most* to the *OC* or *Var*(*N*) of P.

For the current pattern in the list, we randomly select a match position *i* and a don’t-care position *j*. If swapping *i* and *j* does *not* improve the current pattern set, we move on to the next pattern in the list and proceed in the same way. This is repeated until we find a pattern where swapping the selected pair of random positions does improve P. In this case, the modified pattern is accepted, all values *C*_*r*_ are updated, the patterns in P are sorted accordingly, and we start again with the pattern *P*_*r*_ with maximum *C*_*r*_. If we reach the last pattern in the list without obtaining any improvement, we start again with the first pattern, i.e. the pattern with the largest *C*_*r*_, select new random positions *i* and *j* etc. Processing one pattern *P*_*r*_ in this way takes *O*(*m* ⋅ *ℓ*^2^) time, since we look only at one single pair (*i*, *j*) and calculate the *OC* or *Var*(*N*) of the pattern set that would be obtained by swapping *i* and *j* in *P*_*r*_.

The hill climbing is continued until a user-defined number of pairs (*i*, *j*) have been swapped to improve the current pattern set; by default, 25,000 pairs are swapped. If we want to obtain a pattern set with maximal sensitivity, the described hill-climbing procedure is repeated 100 times, and for the pattern set with the lowest *OC* among the 100 obtained pattern sets, the sensitivity is calculated. To calculate the sensitivity, *rasbhari* uses program code from *SpEED*. Again, this whole process is repeated 5,000 times, so for a total of 5,000 pattern sets the sensitivity is calculated during one program run. This is similar to *SpEED*, but in *SpEED* the time-consuming sensitivity calculation is carried out after *one* round of hill climbing. By contrast, we run our faster hill-climbing routine 100 times before we calculate the sensitivity for the *best* pattern set from these 100 runs. The final output of our program is the pattern set with the highest sensitivity from the 5,000 iterations.

The number *m* of patterns and their weight *w* are to be specified by the user. If *Var*(*N*) is to be minimized for alignment-free sequence comparison, all patterns have the same length *ℓ* which is also to be specified by the user. If the sensitivity is to be maximized for database searching and read alignment, better results are achieved if the patterns in P have different lengths. In this case, the maximum and minimum pattern lengths need to be specified. The program then selects lengths *ℓ*_1_, …, *ℓ*_*m*_ that are evenly distributed between these extreme values.

## Results

### Sensitivity in database searching

To evaluate *rasbhari*, we first applied it to generate pattern sets, maximizing the *sensitivity* for database searching and read mapping. For the number *m* and weight *w* of the patterns and for the length *H* and match probability *p* of the homology regions, we used the parameter settings from *SHRiMP2* [43], *PatternHunter II* [38] and *BFAST* [44]. We and compared it to the sensitivity of pattern sets obtained with *Iedera* [45], *SpEED* [40], *AcoSeeD* [46], *FastHC* and *MuteHC* [47] as published by the authors of these programs; the results of this comparison are shown in Table 1. Here, the sensitivity values of *AcoSeeD* are *average* values over 10 program runs reported in [46].

**Table 1:** Sensitivity comparison of different programs.

*w*	*p*	*Iedera*	*SpEED*	*AcoSeeD*	*FastHC*	*MuteHC*	*rasbhari*
***SHRiMP2:*** **4 patterns (*H* = 50)**							
10	0.75	90.6820	90.9098	90.9513	90.7312	**92.6812**	90.9614
	0.80	97.7586	97.8337	97.8521	97.7625	**98.3836**	97.8554
	0.85	99.7437	99.7569	99.7614	99.7431	**99.8356**	99.7618
11	0.75	83.2413	83.3793	**83.4728**	83.3068	83.4127	83.4679
	0.80	94.9350	94.9861	95.037	94.9453	95.0194	**95.0386**
	0.85	99.2189	99.2431	99.2478	99.2250	99.2486	**99.2506**
12	0.80	90.3934	90.5750	90.6328	90.4735	90.5820	**90.6648**
	0.85	98.0781	98.1589	98.1766	98.1199	98.1670	**98.1824**
	0.90	99.8773	99.8821	99.8853	99.8771	99.8836	**99.8864**
16	0.85	84.5795	84.8212	**84.9829**	84.6558	84.8764	84.969
	0.90	97.2806	97.4321	97.4712	97.3556	97.4460	**97.5035**
	0.95	99.9331	99.9388	99.9419	99.9347	99.9424	**99.9441**
18	0.85	72.1695	73.1664	**73.27**	72.9558		73.2209
	0.90	93.0442	93.7120	93.7778	93.6030		**93.78**
	0.95	99.6690	99.7500	**99.7599**	99.7399		99.7557
***PatternHunterII:*** **16 patterns (*H* = 64)**							
11	0.70	92.0708	93.2526		93.0585		**93.4653**
	0.75	98.3391	98.6882		98.6352		**98.7573**
	0.80	99.8366	99.8820		99.8750		**99.8907**
***BFAST:*** **10 patterns (*H* = 50)**							
22	0.85	60.1535	60.8127		60.0943		**60.9919**
	0.90	87.9894	88.5969		88.0426		**88.8005**
	0.95	99.2196	99.3659		99.2923		**99.4099**

Sensitivity of pattern sets in hit-and-extend database searching, calculated with different programs. Parameter settings for the number *m* and weight *w* of patterns, the length *H* of the gap-free homology region between query and database sequences and the match probability *p* in the homology regions, are taken from three popular programs *SHRiMP2, PatternHunter II* and *BFAST*. Sensitivity values from *rasbhari* were calculated using program code from *SpEED*; results of all other programs are taken from their respective publications.

If pattern sets with maximal sensitivity are to be found, and if the lengths *ℓ*_*r*_ of the patterns are small, the run time of *rasbhari* is comparable to *SpEED*. In this case, the most time-consuming step in both programs is to calculate the sensitivity of pattern sets which, by default, is done 5,000 times per program run in each of the two programs. For longer patterns, however, *SpEED* can be much slower since it carries out hill-climbing until a local minimum is reached and, as explained above, each single step in the hill-climbing procedure of *SpEED* takes *O*(*m*^2^ ⋅ *ℓ*^4^) time. In contrast, *rasbhari* terminates this procedure after a given number of iteration steps, and it considers only a limited number of swaps of *match* and *don’t-care* positions in one iteration step.

### Alignment-free phylogeny reconstruction

**Fig 2:**
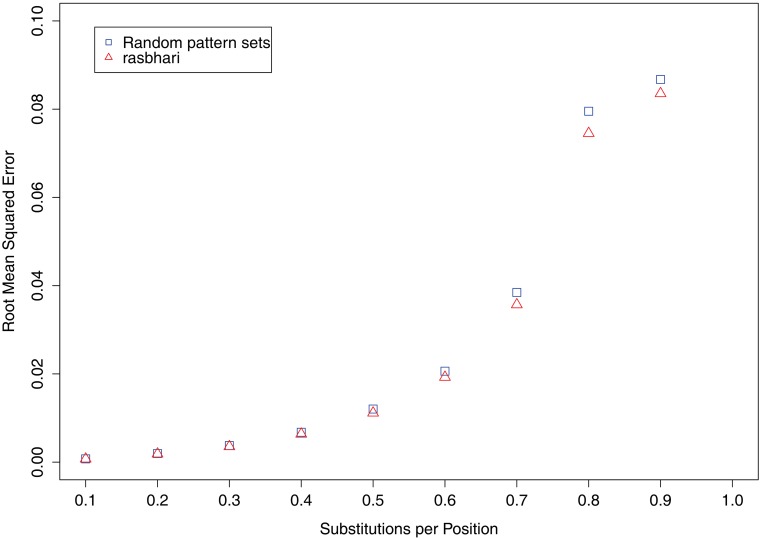
Accuracy of phylogenetic distance estimates based on different pattern sets. Nine sets of DNA sequence pairs were simulated with distances *d* between 0.1 and 0.9 substitutions per position. Distances were estimated based on the number *N* of spaced-word matches between them, using the alignment-free method published in [34]. We used two types of underlying pattern sets, (*a*) pattern sets generated with *rasbhari*, minimizing the variance of *N*, and (*b*) randomly generated pattern sets. The root mean square error of the estimated distances is plotted against the ‘real’ distances *d*.

Next, we wanted to know how alignment-free phylogeny reconstruction can be improved with *rasbhari*. To this end, we simulated pairs of DNA sequences and estimated the distances between them using the *Spaced Words* approach described in [34]. We then measured the accuracy of the distance estimates for different underlying pattern sets. First, we used *rasbhari* to minimize the *variance* of the number *N* of spaced-word matches between two sequences. Since there is no other method to minimize *Var*(*N*), we compared the pattern sets from *rasbhari* with the randomly generated pattern sets that we previously used. The phylogenetic distances estimated with both types of pattern sets were compared to the ‘real’ distances between the sequences, i.e. the average number of substitutions per position. As test data, we generated nine data sets with 2,500 pairs of DNA sequences of length 100 *kb* each. The distances *d* of the sequence pairs ranged between 0.1 and 0.9 substitutions per position. For each program run, we used a set of *m* = 3 patterns of length 20 with 16 *match* and 4 *don’t-care* positions. Fig 2 shows the root mean square error of the estimated distances, compared to the ‘real’ distances *d*. The pattern sets generated with *rasbhari* were superior to the randomly generated pattern sets, especially for large distances.

### Read classification with *CLARK-S*

As a third test case, we used different pattern sets for *CLARK-S* [18, 48], a recently developed tool for short read classification. We evaluated the accuracy of *CLARK-S* with three underlying pattern sets, namely **(A)** with the patterns used by default in the program, **(B)** with patterns from *rasbhari* minimizing *overlap complexity* and **(C)** with patterns from *rasbhari* maximizing *sensitivity*.*CLARK-S* uses sets of *m* = 3 patterns of length *ℓ* = 31 and with a weight of *w* = 22. Since *SpEED* is too slow to generate pattern sets with long patterns, the authors of the program generated pattern sets for *CLARK-S* by exhaustively searching over all *single* patterns with *ℓ* = 31 and *w* = 22. If the first and the last position in the patterns are required to be *match positions*, this approach has to evaluate $\left(\begin{array}{c} 29\\ 20 \end{array}\right)\approx 10^{7}$ possible patterns. The sensitivity of each of these patterns was calculated, and the three patterns with the highest sensitivity were selected. Note however, that maximizing the sensitivity of *single* patterns is only an approximation to finding a *set* of patterns with maximal *total* sensitivity.

**Fig 3:**
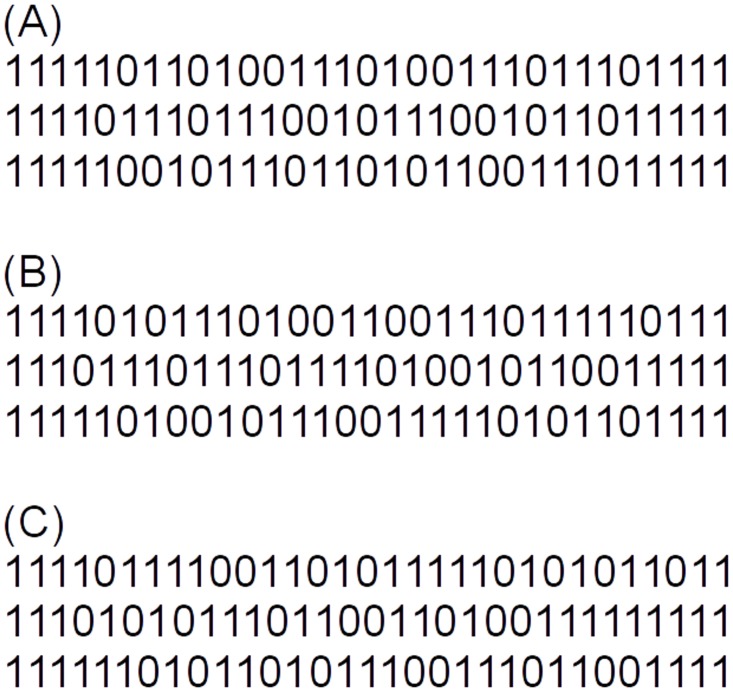
Pattern sets for short read classification. Pattern sets used for short read classification: **(A)** as used by default in *CLARK-S*, **(B)** generated with *rasbhari* minimizing *overlap complexity* and **(C)** generated with *rasbhari* maximizing *sensitivity*.

Fig 3 shows the default pattern set from *CLARK-S* and the two pattern sets generated by *rasbhari* as described. The exhaustive procedure used by *CLARK-S* took 2 hours to generate the pattern set.*rasbhari*, by contrast, calculated pattern sets with the same parameters within 7.54 seconds with the *slow* version where the *sensitivity* is calculated, and within 0.068 seconds with the *fast* version where the *overlap complexity* is maximized without considering the sensitivity explicitly. The slow version of *rasbhari* is thus around 480 times faster than the exhaustive procedure in *CLARK-S*, while the fast version is around 52,000 times faster. The theoretical sensitivity of the three pattern sets is 0.999771 for the default patterns from *CLARK-S*, 0.999811 for the *rasbhari* patterns with minimized overlap complexity and 0.999822 for the *rasbhari* patterns with maximized sensitivity.

To evaluate the classification accuracy of *CLARK-S* with these three pattern sets experimentally, we used five data sets from the literature, namely two sets, *HC1* and *HC2*, from the *MetaPhlAn* project [49] and three sets, *simHC, simMC and simLC*, from the *FAMeS* databases [50]. For each of these data sets, we calculated *precision* and *sensitivity* of the classification at the species level as defined in [11]. That is, for a classification task where objects are to be assigned to classes, *precision* is defined as the fraction of correct assignments among the total number of assignments, while *sensitivity* is the ratio between the number of correct assignments and the number of objects to be classified. The two values are not the same since not every object is necessarily assigned to one of the classes; *precision* is always larger than or equal to *sensitivity* since the denominator in the definition of precision is smaller or equal to the denominator in the definition of sensitivity. Since this definition of *sensitivity* refers to the ability of a program to correctly classify objects, it is not to be confused with the sensitivity in database searching as discussed above. Table 2 summarizes precision and sensitivity of *CLARK-S* with its default pattern set and with a pattern set generated by *rasbhari*.

**Table 2:** Read classification with CLARK-S using different pattern sets.

		Default pattern set		rasbhari	
Dataset	#reads	Precision	Sensitivity	Precision	Sensitivity
HC1	999,998	97.69	90.36	97.69	**90.44**
HC2	999,991	96.45	88.11	96.45	**88.18**
simHC	116,771	97.20	90.53	97.20	**90.54**
simMC	97,495	**98.75**	95.09	98.73	95.09
simLC	114,457	**98.29**	**94.26**	98.28	94.25

Read classification with *CLARK-S* [18] with the default pattern set of the program and with the pattern set from *rasbhari* for the same parameter values, namely *n* = 3 patterns of length *ℓ* = 31 and weight *w* = 21. *Precision* and *sensitivity* of the classification are reported at the *species level* for five data sets from the literature.

**Fig 4:**
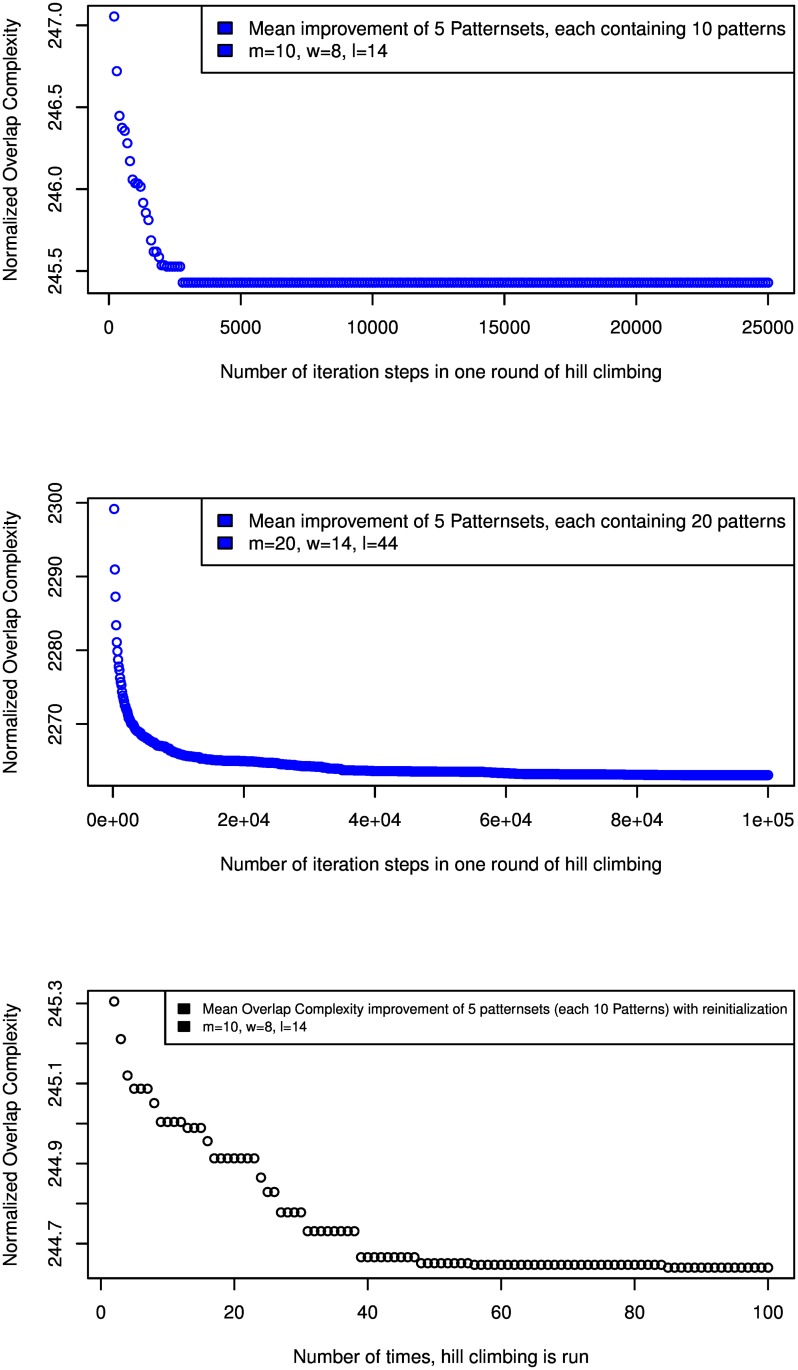
overlap complexity of pattern sets in the hill-climbing algorithm. Normalized overlap complexity *(OC)* of pattern sets depending on the number of iteration steps in our algorithm. The first two plots show how the *OC* is reduced in a single round of the hill-climbing algorithm for different parameters. For a set of *m* = 10 patterns of length *ℓ* = 14 and weight *w* = 8, the algorithm converges after around 3,000 iteration steps of hill-climbing (upper plot); for a set of *m* = 20 patterns of length *ℓ* = 44 and weight *w* = 14, it converges after around 80,000 steps (middle plot). The lower plot shows how the *OC* is improved if the hill-climbing algorithm is run multiple times and the best result of all runs is returned.

Fig 4 shows how the overlap complexity *(OC)* of pattern sets produced by *rasbhari* depends on the number of iteration steps carried out in the hill-climbing algorithm. For a set of *m* = 10 patterns of length *ℓ* = 14 and weight *w* = 8, a single run of the hill-climbing procedure converges after around 3,000 steps; for *m* = 20, *ℓ* = 44, *w* = 14, it converges after around 80,000 steps. The *OC* is further improved if the hill-climbing procedure is run multiple times and the best result of these runs is used.

In the previous section, we mentioned that the *OC* is related to the variance of the number *N* of spaced word matches. Comparison of eqs (5) and (2) showed that, in the special case where *p* = 1/2 and the contribution of the ‘background’ spaced-word matches is small, minimizing the *OC* is equivalent to minimizing the variance of *N*. In general, however, this is not the case, as the following example shows. We applied *rasbhari* to generate two sets of *m* = 10 patterns each, with length *ℓ* = 20 and weight *w* = 8, one set by minimizing the *OC* and the other one by minimizing *Var*(*N*). When generating the second set, we used a match probability of *p* = 0.75 and a sequence length of *L* = 10,000. The pattern set that we obtained when we minimized the *OC* had an *OC* of 11,116, the set for which we minimized *Var*(*N*) had an *OC* of 11,195. Conversely, when we minimized *Var*(*N*), we obtained a pattern set with a variance of 156,061, while the variance was 156,152 when we minimized the *OC*. It thus makes a difference which one of these two parameters is minimized.

## Discussion

We developed a program called *rasbhari* to calculate sets of binary *patterns*—or *spaced seeds*, as they are often called—for read mapping, database searching and alignment-free sequence comparison. For sequence-homology searching, *rasbhari* optimizes the *sensitivity* of pattern sets, i.e. the probability of obtaining at least one hit between a query and a database sequence sharing a gap-free homology of a given length and with a given match probability between nucleotides. Since the sensitivity of a pattern set is expensive to calculate, our algorithm optimizes the *overlap complexity* of the produced pattern sets which is closely related to its sensitivity. We use a hill-climbing algorithm, similar to the one used in *SpEED*, to minimize the overlap complexity. Unlike *SpEED*, however, our algorithm does not calculate the overlap complexity of *all* neighbours of a current pattern set, but modifies those patterns first that contribute most to the overlap complexity of the current pattern set. To maximize the sensitivity in database searching, we calculate the sensitivity of the current pattern set after a certain number of iterations. We repeat this procedure and, finally, we pick the pattern set with the highest sensitivity in all iterations.

Since calculating the sensitivity is time consuming, *rasbhari* can alternatively minimize the overlap complexity alone, without calculating the sensitivity of pattern sets. This option is useful in situations where large pattern sets are needed for which it would take too long to calculate the sensitivity. As a third option, *rasbhari* can minimize the variance of the number *N* of spaced-word matches in alignment-free sequence comparison which is used by various methods to estimate phylogenetic distances between sequences. We could show that, mathematically, the variance of *N* has a similar form as the overlap complexity of a pattern set, so the same optimization algorithm can be used to minimize both of them.

In both homology searching and read classification, pattern sets generated by *rasbhari* are more sensitive than alternative pattern sets, so more homologies can be detected and more reads can be correctly classified. At first glance, the increase in sensitivity that we obtained seems moderate; as shown in Table 1, the improvement is usually in the first or second digit after the decimal mark. In database searching and read mapping, however, even small improvements in sensitivity can lead to a large number of additional hits. Moreover, as these additional hits will be mostly in the ‘twilight zone’ of low sequence similarity, they may be of particular interest to the user.

In the context of read alignment, Ilie et al. pointed out that, with a 100-fold coverage of the human genome, a 1 percent improvement in pattern sensitivity would mean that 3 billion more nucleotides could be mapped [40], so the improvement that we achieved with *rasbhari* would still lead to tens or hundreds of millions of additionally mapped nucleotides. In database searching, the situation is similar. If we consider, for example, homology regions of length *H* = 64 with a match probability of *p* = 0.8 at the nucleotide level, then with *w* = 11, the sensitivity of *rasbhari* is improved by less than 0.01 percentage points compared to *SpEED*, see Table 1. Note, however, that these sensitivity values are already close to 100%, so the fraction of homologies that are *not* detected can be considerably reduced with the slight improvement in sensitivity obtained with *rasbhari*. In our example, the number of homologies that are *missed* is reduced by >7% if *rasbhari* is used instead of *SpEED*. With the same parameters, but with *p* = 0.7, the sensitivity of both programs is around 93%. Here, the number of missed homologies is still reduced by 3% with *rasbhari*, compared to *SpEED*.

For alignment-free sequence comparison, pattern sets produced by *rasbhari* lead to more accurate phylogenetic distances than the random pattern sets that we previously used. While this result may not be surprising, *rasbhari* is, to our knowledge, the first program that has been designed for this purpose and that can minimize the variance of the number of spaced-word matches. We therefore integrated *rasbhari* into our web server for alignment-free sequence comparison [41].

In read classification, the sensitivity of *CLARK-S* could be increased by 0.08 and 0, 07 percentage points, respectively, for the largest data sets that we used, *HC1* and *HC2*. Each of these data sets contains around one million reads, so the improvement in sensitivity that we achieved with *rasbhari* means that 800 more reads from *HC1* and 700 more from *HC2* could be correctly classified by *CLARK-S*. This is remarkable, since the classification accuracy of *CLARK-S* is already very high, so it is hard to further improve the program. An interesting question in the context of *CLARK-S* is how the length and weight of the patterns influence its accuracy. So far, it was difficult to investigate this question systematically, since the exhaustive method that the program uses by default, is too time consuming. With the massive improvement in runtime that we achieved with *rasbhari*, it is now possible to systematically investigate how the accuracy of *CLARK-S* depends on the parameters of the underlying pattern sets.

In the hill-climbing procedure, our default of 25,000 iteration steps was sufficient to obtain stable results for the parameter settings that we used in our benchmark studies; we were unable to further improve these results by increasing the number of iterations. For different values of *m*, *w*, *ℓ*, *p* and *H*, however, it may be advisable to adapt the number of iteration steps. Fig 4 shows that, if the number of patterns or their length and weight are increased, a larger number of iteration steps can improve the results. The number of iterations within one round of hill climbing and the number of times the hill-climbing is carried out can be passed to *rasbhari* through the command line; the users can therefore adapt these parameter values for their particular applications if they do not want to use the default values of the program.
